# Can the Diagnostics of Triangular Fibrocartilage Complex Lesions Be Improved by MRI-Based Soft-Tissue Reconstruction? An Imaging-Based Workup and Case Presentation

**DOI:** 10.1155/2017/5870875

**Published:** 2017-01-29

**Authors:** Niels Hammer, Ulrich Hirschfeld, Hendrik Strunz, Michael Werner, Thomas Wolfskämpf, Sabine Löffler

**Affiliations:** ^1^Department of Anatomy, University of Otago, Dunedin, New Zealand; ^2^Department of Anatomy, University of Leipzig, Leipzig, Germany; ^3^Department of Orthopedic and Trauma Surgery, University Clinics of Leipzig, Leipzig, Germany; ^4^Fraunhofer Institute for Machine Tools and Forming Technology (IWU), Dresden, Germany

## Abstract

*Introduction*. The triangular fibrocartilage complex (TFCC) provides both mobility and stability of the radiocarpal joint. TFCC lesions are difficult to diagnose due to the complex anatomy. The standard treatment for TFCC lesions is arthroscopy, posing surgery-related risks onto the patients. This feasibility study aimed at developing a workup for soft-tissue reconstruction using clinical imaging, to verify these results in retrospective patient data.* Methods*. Microcomputed tomography (*μ*-CT), 3 T magnetic resonance imaging (MRI), and plastination were used to visualize the TFCC in cadaveric specimens applying segmentation-based 3D reconstruction. This approach further trialed the MRI dataset of a patient with minor radiological TFCC alterations but persistent pain.* Results*. TFCC reconstruction was impossible using *μ*-CT only but feasible using MRI, resulting in an appreciation of its substructures, as seen in the plastinates. Applying this approach allowed for visualizing a Palmer 2C lesion in a patient, confirming ex postum the arthroscopy findings, being markedly different from MRI (Palmer 1B).* Discussion*. This preliminary study showed that image-based TFCC reconstruction may help to identify pathologies invisible in standard MRI. The combined approach of *μ*-CT, MRI, and plastination allowed for a three-dimensional appreciation of the TFCC. Image quality and time expenditure limit the approach's usefulness as a diagnostic tool.

## 1. Introduction

The proximal wrist plays an important role in activities of daily life, allowing for a wide range of motion of the hand and simultaneously providing stability [1–4]. The triangular fibrocartilage complex (TFCC)[5], composed of cartilage and ligaments situated between the distal radius and ulna, the triquetrum, and lunate, plays an integral part in load transfer between the forearm and hand at the various positions of the hand [3]. TFCC lesions are likely caused by trauma or overstrain. Due to the complexity of the TFCC [6, 7] and the variety of potential lesions, image-based diagnosis is often difficult [8, 9], which has implications for daily life and work ability of the affected patients [10]. Clinical visualization by computed tomography (CT) [11] or magnetic resonance imaging (MRI) [12, 13] has certain disadvantages concerning the achievable resolution and soft tissue differentiation to the effect that a distinct and reliable diagnosis is often hampered. To date, the gold standard for reliably diagnosing TFCC lesions is wrist arthroscopy [14]. The invasiveness of this procedure however underlines the necessity for less invasive diagnostic tools to minimize surgery-related risks for the patient [15]. Three-dimensional reconstructions on the basis of clinical imaging have repeatedly been proven to be of diagnostic value for joints and muscles [16–18]. However, to the authors' best knowledge, no attempt has yet been made to implement such reconstructions as a diagnostic tool for TFCC pathology.

The aim of the given technical feasibility study and case report was to investigate whether such three-dimensional reconstructions may also help to visualize the TFCC on the basis of clinical imaging and to critically evaluate whether such reconstructions can be used as a diagnostic tool.

## 2. Materials and Methods

### 2.1. Postmortem Examinations including *μ*-CT, MRI, and Plastination

Two TFCCs were obtained postmortem from one human cadaver (male, 81 years). In a supination position of the hand, the styloid process of the ulna, Lister's tubercle, the capitate, and the pisiform were identified as bony landmarks. After removal of a rectangular skin flap, the fourth to sixth extensor compartments were identified. The respective tendons were transected at the level of the midcarpal joints. Following this, the TFCCs were removed en bloc using three additional incisions: one distal of the pronator quadratus transecting the radius and ulna, one in the midcarpal joint line, and one lateral of the lunate.

Clinical imaging of the TFCCs was carried out using combined *μ*-CT, 3 T MRI and plastination. For this purpose, the TFCCs were embedded in 10 mass% gelatin under vacuum to minimize potential artifacts. The specimens were scanned using a General Electric phoenix v/tome/x s *μ*-CT (GE Measurement & Control Solutions, Wunstorf, Germany) with an isovoxel size of 70 *μ*m. The TFCCs were then scanned using a Magnetom Tim Trio-Scanner (Siemens Medical Solutions, Erlangen, Germany). T2-weighted sequences [19] were obtained using the following parameters: repetition time TR = 13.95 ms, echo time = 5.20 ms, slice thickness = 0.4 mm, Field of view = 87.5 × 100 mm, matrix = 448 × 512 pixels, and acquisition time of 6 minutes. For plastination, the two TFCCs were shock frozen in 85% acetone at −85°C [20] and then dehydrated for two weeks in ascending acetone series. The specimens were then force-impregnated according to the standard procedure [21] using E1/E10/E12 (Biodur, Heidelberg, Germany) at a ratio of 28/10/100. Following the curing in a heating chamber, serial sections with a thickness of 250 *μ*m were obtained from the four blocks. Two blocks were cut with a diamond ring saw (DRAMET GmbH, Kleinmaischeid, Germany) in the coronal plane, two in the sagittal plane. The connective tissues were stained using methylene blue and parafuchsin [22], and the bone was counterstained using acetone-diluted alizarin [20].

### 2.2. Image Segmentation

Segmentation was considered as the process of highlighting structures of interest in imaging datasets, resulting in three-dimensional volumes of the highlighted anatomical structures. For this purpose, Materialise Mimics (Leuven, Belgium) was utilized. Both relevant soft and hard tissues including the TFCC and the surrounding soft tissues were segmented in the cadaveric tissue as well as in the clinical datasets of the patients with a suspected TFCC pathology.

### 2.3. Patient Dataset

One clinical case was investigated of a 31-year-old male patient with a suspected TFCC pathology following hand injury. This case included clinical examination findings, corresponding X-ray, and MRI scans as well as records from hand arthroscopy.

## 3. Results

### 3.1. Three-Dimensional Reconstruction of the TFCC on the Basis of *μ*-CT, MRI, and Plastination

**Figure 1:**
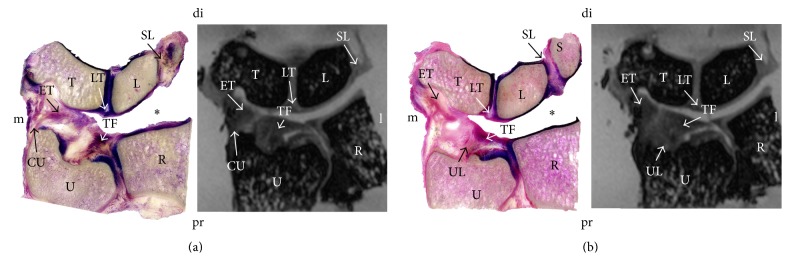
Plastination of the triangular fibrocartilage disc complex. Dorsal coronal section (a) and ventral coronal section (b) in a similar plane and orientation for plastination (left) and magnetic resonance imaging (right). Note the fibrocartilaginous attachments of the complex at the radius, ulna, and triquetrum. The asterisk (*∗*) indicates a deformation artifact introduced due to dehydration-related deformation. CU = collateral ulnar ligament; DRU = dorsal radioulnar ligament; ET = extension of the triangular fibrocartilage; LT = lunotriquetral ligament; SL = scapholunate ligament; TD = triangular disc; TF = triangular fibrocartilage; UL = ulnolunate ligament; L = lunate; R = radius; S = scaphoid; T = triquetrum; U = ulna; di = distal; l = lateral; m = medial; pr = proximal.

**Figure 2:**
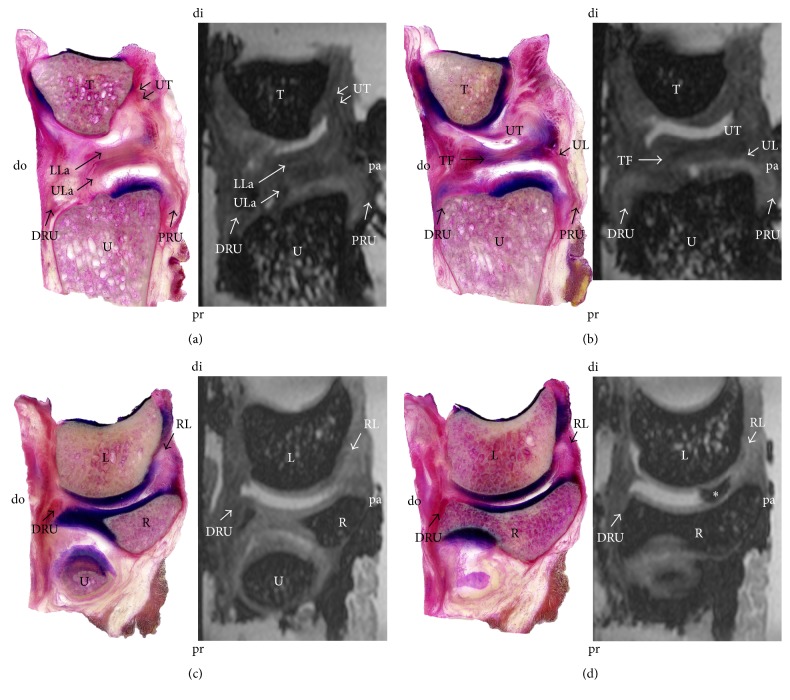
Plastination of the triangular fibrocartilage disc complex. Sagittal section, from medial (a) to lateral (d) in a similar plane and orientation for plastination (left) and magnetic resonance imaging (right). Collagens are stained in red; cartilage is in blue. Note the anchoring of the TFCC at the distal ulna (a) and the fibrous continuity of the complex (b–d). (a, b) DRU = dorsal distal radioulnar ligament; LL = lower lamina^#^; PRU = palmar distal radioulnar ligament; TF = triangular fibrocartilage; UL = ulnolunate ligament; UL = upper lamina^#^; UM = ulnomeniscal homologue; UT = ulnotriquetral ligament; T = triquetrum; U = ulna; d = dorsal; di = distal; pa = palmar; pr = proximal. (c, d) DRU = dorsal distal radioulnar ligament; RL = radiolunate ligament; L = lunate; R = radius; U = ulna; d = dorsal; di = distal; pa = palmar; pr = proximal. ^#^Nomenclature adapted from Benjamin et al. [23].

**Figure 3:**
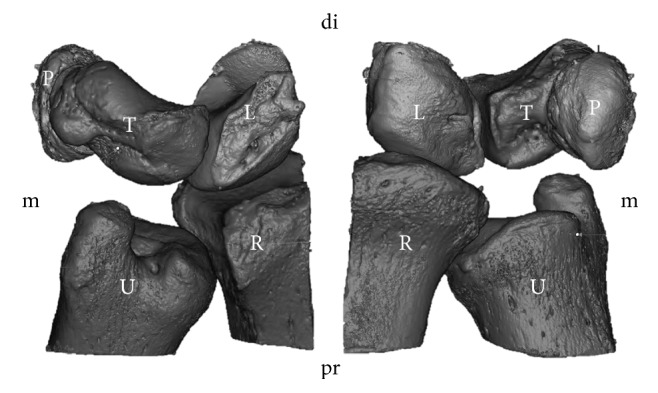
Image-based reconstruction from microcomputed tomography and magnetic resonance imaging. The triangular fibrocartilage complex could not be visualized to sufficient extent using this method.

**Figure 4:**
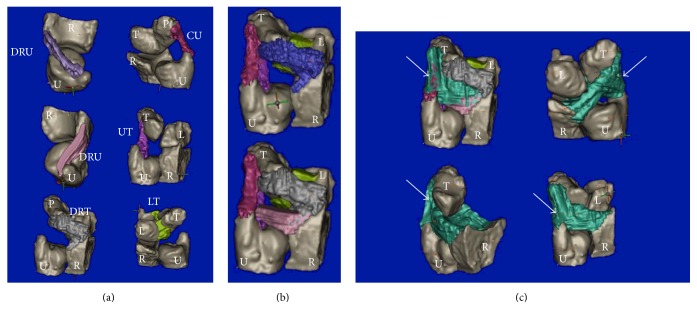
Segmentation-based three-dimensional reconstruction of the triangular fibrocartilage complex substructures. CU = collateral ulnar ligament; DRT = dorsal radiotriquete ligament; DRU = dorsal radioulnar ligament; L = lunatum; LT = lunotriquetral ligament; P = pisiform; R = radius; T = triquetrum; U = ulna; UT = ulnotriquetrum ligament. (a) Top left: distal view; top right: lateral view; middle left: distal view; middle right: dorsal view; bottom left: dorsal view; bottom right: palmar view. (b) Dorsal view: top, without smoothening; bottom, with smoothening. (c) Entire triangular fibrocartilage complex (arrow). Top left: dorsal view; top right: palmar view; bottom left: lateral view without lunate; bottom right: dorsal view.

In 3 T MRI and in plastination, the TFCCs were visualized in situ, allowing for a detailed section-based differentiation of the soft tissues.  Figure 1 gives an overview in the coronal plane and Figure 2 in the sagittal plane. In the stained samples, the origin *μ*-CT resulted in a superior visualization of the distal radius, ulna, and the medial carpals (Figure 3). However, the soft tissues were hardly differentiable in *μ*-CT. The image-based reconstruction of the MRI datasets helped to visualize the structures of the TFCC in more detail, thereby providing a three-dimensional overview of the fibrocartilage and adjacent ligaments.  Figure 4 summarizes the individual structures the TFCC and the spatial alignment.

### 3.2. Ex Postum Validation with a Patient Case

**Figure 5:**
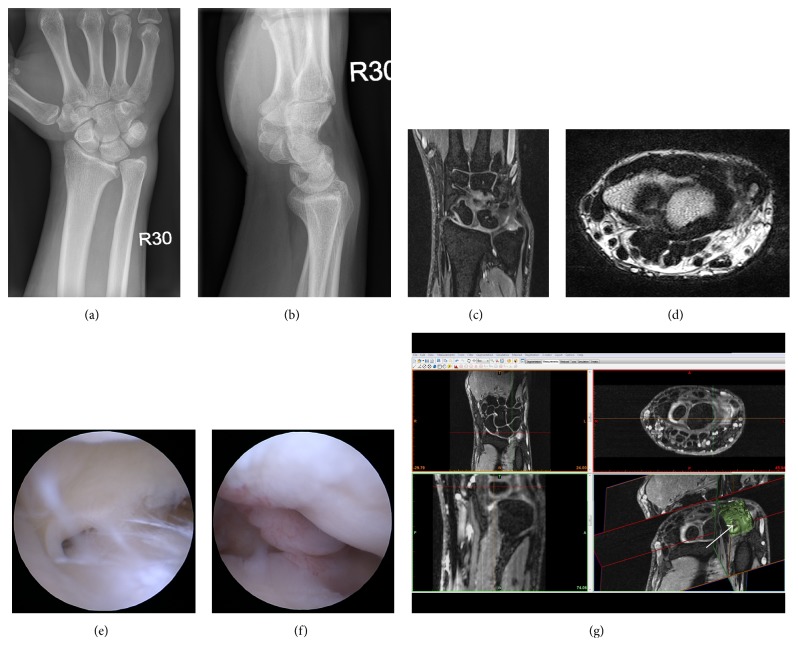
Case of a 31-year-old male. (a, b) X-ray of the right wrist: anterior-posterior (a) and lateral (b) view. (c, d) T2-weighted magnetic resonance imaging in the sagittal (c) and coronal (d) plane, suspected diagnosis Palmer 1B lesion. (e, f) Hand arthroscopy of the same patient, showing a Palmer 2C lesion (e) and injected synovial villi at the radioscapholunate ligament (f). Figure 5(g) shows all anatomical planes in 1.5 T magnetic resonance imaging (top row, bottom left) and a three-dimensional reconstruction of the ulnocarpal disc region (bottom right) of the same patient. The defect cannot be visualized clearly in the standard planes from the MRI. The arrow points at the lesion in the ulnocarpal disc in the segmentation-based reconstruction, indicated by the flattened groove surrounded by two bulky tissue remainders on either side.

The X-rays taken at the day of trauma and subsequently Figure 5(a) and Figure 5(b) did not show any evidence for osseous lesions. Having persistent pain and related restrictions of hand use for more than twelve weeks under continuous physiotherapy, an MRI was taken, showing an ulnar avulsion of the TFCC without any malpositioning, classified as a Palmer 1B lesion (Figure 5(c) and Figure 5(d)). Due to further pain persistence under continuous physiotherapy, an indication for hand arthroscopy was set. The patient demonstrated a perforation of the ulnar disc (Palmer 2C, Figure 5(e)), which free ends, together with villi (Figure 5(f)), were removed by shaving debridement. In the seven months of follow-up after the initial trauma and two months after the surgical intervention with subsequent physiotherapy, the patient recovered fully, reporting only minor pain in a few instances. In accordance with the findings from hand arthroscopy, the three-dimensional reconstruction of the TFCC revealed a central perforation at the TFCC, as indicated in Figure 5(g). This perforation was however not seen in the standard planes from MRI.

## 4. Discussion

### 4.1. MRI-Based Segmentation with Three-Dimensional Reconstruction May Add Diagnostic Value for TFCC Pathology: Limitations Apply

Image-based diagnosis of the TFCC is made difficult by the complexity of the TFCC [5] and the variety of potential lesions [7, 9, 12, 14, 24]. There is sound evidence for the TFCC to be both a structure of load transfer between the forearm and hand [1, 4, 10, 24–26] and likewise an organ of mechanoreception and proprioception [27]. Diagnosis on the basis of clinical imaging is, however, limited for the TFCC [28], in spite of new approaches using ultrasound [29] and MRI-arthrography techniques with [30] or without finger trap distraction [31–33]. Higher resolution MRI may help to partly resolve this issue [34]. However, such scanners are to date only available to limited extent for the clinical routine.

The given feasibility study is the first to use 3 T MRI as a basis for TFCC reconstruction. From our results, *μ*-CT may not serve well to visualize the TFCC structures without further contrasting agents, as done by De Filippo and coworkers [35] and Moser et al. [36]. Moreover, due to the complexity of the TFCC, it is challenging to differentiate potential lesions of the substructures. One option to enhance the informative value of clinical imaging is to create three-dimensional reconstructions of the datasets [37]. This is particularly difficult for inhomogeneous substructures as in the TFCC, consisting of cartilage as well as surrounding ligaments and tendons [26]. Comparison of the cadaveric datasets to the clinical data used in this study already points out the advantages of higher resolution imaging, as shown for other anatomical structures previously [38]. The given study has shown that even if standard MRI shows negative results or minor structural impairments, as shown for the TFCC here, a significant pathology might still exist, confirming recent reports [14]. Such pathologies might be recognized in three-dimensional reconstructions of the TFCC on the basis of clinical imaging.

A number of limitations exist for the given study and the general framework regarding diagnostic TFCC reconstruction on the basis of segmenting clinical datasets. First, the segmentation procedure itself is accompanied by a learning curve to the effect that only a three-dimensional understanding of the spatial relations of the TFCC substructures helps to segment these tissues properly. Second, the segmentation itself largely depends on the quality of the MRI imaging; that is, lower resolution scans might not provide sufficient information. Third, it needs to be taken into account that the results presented here were based on an individual dataset and pathology and that the diagnosis from the three-dimensional reconstruction was done ex postum to the effect of a potential bias by the person carrying out the segmentation, clearly making these findings preliminary as part of a technical feasibility study. Additionally, the costs related to the extended MRI scans, the additional workload for the manual segmentation, and the software have to be taken into account, though there is an amount of freeware available for this purpose. Arthroscopy has the large advantage to serve as a combined diagnostic and therapeutic tool [39], which is not the case using three-dimensional reconstructions.

It can be concluded that image-based three-dimensional reconstruction of the TFCC substructures may provide additional information on TFCC pathology on the basis of MRI, potentially to the effect of a more precise diagnosis and preoperative planning. At this stage, however, this method may unlikely replace the gold standard of wrist arthroscopy due to the given limitations and lacking case numbers. Clinical application has to be seen critically. However, this approach has some implications and a didactic value. Future developments to (semi)automatically identify the TFCC might improve the attractiveness of this procedure.
